# Complex upper limb reconstruction using dorsoepigastric flap. Case report of a convenient resource

**DOI:** 10.1016/j.ijscr.2019.08.002

**Published:** 2019-08-09

**Authors:** Gustavo Jimenez Muñoz-Ledo, Marcos Melgosa-Juárez, Julio Palacios-Júárez, Jesus Morales-Maza, Jorge Humberto Rodríguez-Quintero

**Affiliations:** a Plastic and Reconstructive Surgery Department, Hospital Aranda de la Parra, Guanajuato, Mexico; b Orthopedic Surgery Department, Hospital Aranda de la Parra, Guanajuato, Mexico; c Plastic and Reconstructive Surgery Department, Hospital Regional de Alta especialidad Ixtapaluca, Mexico City, Mexico; d Surgery Department, Instituto Nacional de Ciencias Médicas y Nutrición “Salvador Zubirán”, Mexico City, Mexico

**Keywords:** Dorsoepigastric flap, Arm reconstruction, Mangled extremity, Reconstructive surgery

## Abstract

**Introduction:**

Dorsoepigastric flap (DF), first described by. Haddad and Jimenez, is a variant of the classic lattisimus dorsi musculocutaneous flap that only utilizes a minimum quantity of muscle tissue, through which the vascular pedicle passes by. It has been used primarily as an acceptable alternative in mammary reconstruction when the use of thoraco abdominal muscles is not viable, and offers several advantages such as adequate flap volume with generous cutaneous island dimensions and functional preservation of the latissimus dorsi.

**Presentation of a case:**

We report the case of a 12 year old male, who suffered a left upper limb injury secondary to high power firearm shot. He presented extensive damage to the skin, soft tissues and bone structures and was treated with primary reconstruction using DF and peroneal grafts. Three years later, a shoulder prosthesis was placed to improve limb function with unobjectionable results.

**Conclusion:**

DF is a useful resource that has been merely used in the context of complex upper limb reconstruction, and must be considered essential in the repertoire of the reconstructive surgeon when facing traumatic defects of the upper extremity.

## Highlights

• Dorsoepigastric flap is a variant of the classic lattisimus dorsi flap that utilizes less muscle tissue.
• It is an appropriate reconstructive alternative and has the advantage of functional preservation of the lattisimus dorsi muscle.
• It must be considered as part of the repertoire of the reconstructive surgeon when fixing complex defects of the upper limb.

## 1 Introduction

Complex upper limb injuries include a wide spectrum of lesions commonly encountered in the setting of work-related/ motor vehicle accidents as well as in combat and confrontations, due to the instinctive use of the arms when facing dangerous situations. Initial approach to this patients commonly occurs in the accident scene, and must focus on implementing live saving maneuvers such as stopping hemorrhage by means of local compression and tourniquet application, as well as splinting and transporting the patient to an appropriate trauma center where advanced reanimation and volume reposition with IV fluids and blood products can be carried out accordingly. An early notification should be made to all involved medical specialties, commonly including general, vascular, orthopedic and reconstructive surgeons, as well as all the nonsurgical specialties which may play a critical role in the treatment and stabilization of the patient.

Once stable, a conjoined decision should be made between all involved professionals, the patient and his family, in terms of whether a limb-saving procedure should be attempted. The decision must be made based on the available resources (both human and material), the mechanism of trauma, and the status of the extremity and remaining viable structures at the time of evaluation. Current evidence supports that a primary definitive reconstructive procedure results in improved functional and aesthetic outcomes for this individuals.

Appropriate debridement and lavage of contaminated and nonviable tissue must be initially performed as needed depending on the particular characteristics of the case via a formal surgical revision. Attention must be paid to not performing extensive/unnecessary debridement, and the decision should be made based on the consistency, color, contamination and bleeding of tissues during exploration. Status of the remaining viable muscular tissue is the main predictor of the functionality the patient will have after a reconstructive procedure or amputation is carried out. If extensive damage and contamination to soft tissues is observed along with the presence of complex fractures, definitive reconstruction may be postponed. Other factors that must be evaluated, but are not determining for deciding amputation, is the status of vascular and nervous tissues in the injured extremity.

There are several tools, that have been utilized as objective parameters to guide clinical decisions in such catastrophic events, because of all the implications (including medical/legal) an unfavorable outcome could represent; However, its application has not shown conclusive or beneficial results in this setting. On the other side, it is important to consider that a well performed amputation will be crucial for preserving functional outcomes after limb rehabilitation, and that it must be carried out on a timely manner when indicated to improve prognosis and avoid putting the patient at risk of other complications.

Dorsoepigastric flap (DF), first described by Dr. Haddad and Jimenez, is a variant of the classic latissimus dorsi musculocutaneous flap that only utilizes a minimum quantity of muscle tissue, through which the vascular pedicle (the descending branch of thoraco orsal artery) passes by. It has been used primarily as an acceptable alternative in mammary reconstruction when the use of thoracoabdominal wall muscles is not viable, and it offers several advantages such as adequate flap volume with generous cutaneous island dimensions and preservation of functionality by sparing latissimus dorsi and rectus abdominis muscles. DF is a useful resource, which has been merely used in the context of complex arm trauma. Its use must be considered essential in the repertoire of the reconstructive surgeon when facing complex traumatic defects of the upper extremity.

This work has been reported in line with the SCARE criteria [1]. Written informed consent was obtained from the patient´s parents for publication of this case report and accompanying images.

## 2 Case report

We present the case of a twelve year old male patient, with no relevant past medical history, who suffered a high power fire weapon injury to his left upper arm during a street armed robbery. He presented extensive loss of soft tissues, including part of the proximal third and midshaft of the humerus, part of the biceps muscle, and complete loss of subcutaneous fat and skin over the posterolateral aspect of the arm. He was assisted by local paramedics who applied a splint and proximal tourniquet to the extremity and initiated hydric reanimation during transportation to our third level trauma center. At arrival, the patient was found hemodynamically stable, with a grossly contaminated wound over the aforementioned location, so a formal surgical exploration was indicated.

**Fig. 1:**
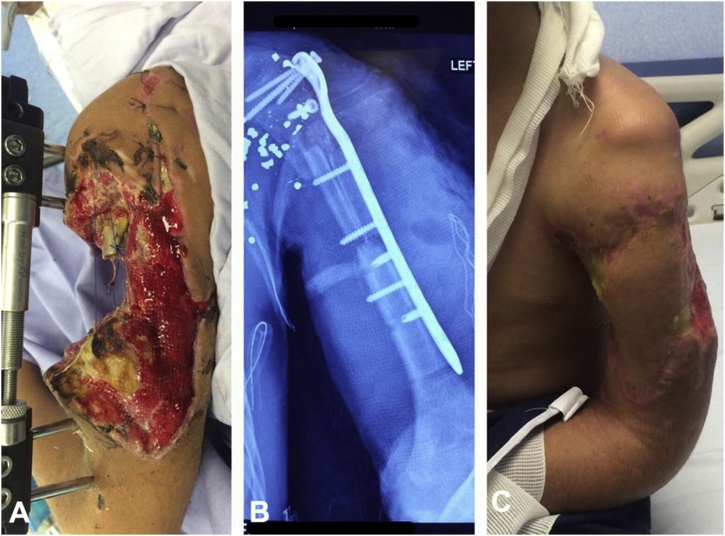
A) Posterior view of the left upper extremity after initial debridement and external fixation. Notice the extensive loss of bone and muscular tissue from the upper arm. B) Post operatory anterior posterior X ray of the left upper extremity displaying bone grafts fixated with a metal plate to the remaining bone tissues of the arm. C) Early post operatory image showing a functional extremity with an integrated DF.

**Fig. 2:**
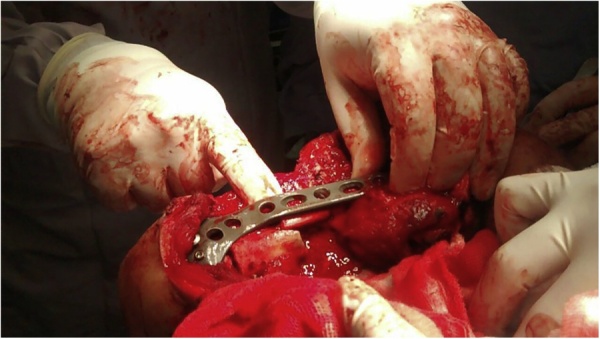
Picture showing the patient in lateral decubitus during primary reconstruction. Peroneal grafts are being used to reconstruct the damaged humerus while being fixated to surrounding viable bone tissue.

**Fig. 3:**
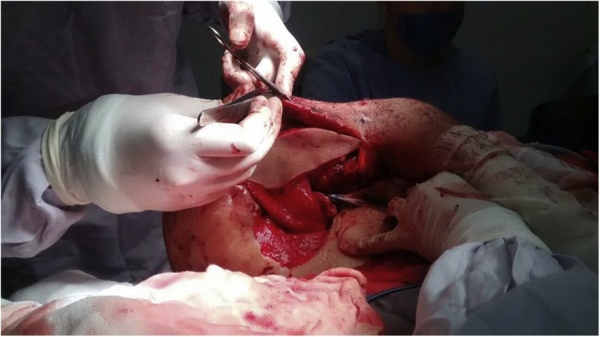
Picture showing the patient in lateral decubitus during primary reconstruction. DF is already dissected and ready to be fixated in the left upper extremity after extensive cleaning and debridement.

During this initial procedure extensive irrigation and debridement of nonviable tissue were performed, followed by external fixation of the humerus, aiming for a definitive reconstructive procedure during a second intervention (Fig. 1A). After broadly explaining the risks and possible outcomes of attempting an extremity saving procedure, the patient and his family decided to accept the operation. During the reconstruction, bilateral peroneal grafts were harvested to reconstruct the damaged bone, and were fixated with a titanium metal plate to the proximal and distal humeral remnants in the upper arm (Fig. 1B). Also, an ipsilateral dorsoepigastric pediculated flap, with a cutaneous island of 18 × 7 cm was designed and utilized for coverage of the defect (Figs. 1C, 2 and 3).

**Fig. 4:**
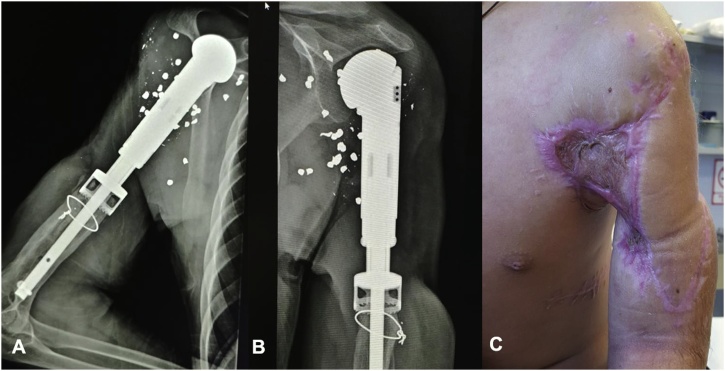
A) X ray taken after total shoulder prosthetic replacement 3 years after the initial procedure. B & C) Pictures taken during patients follow up showing functional and aesthetically acceptable results in his left upper extremity at follow up.

The patient evolved uneventfully, maintaining stability on the upper extremity and was discharged home, presenting appropriate integration of the flap during follow up visits. He attended rehabilitation and gradually reintegrated to his daily activities. Functionally the patient presented suboptimal outcomes, with subtle limitation of the range of movement caused by inadequate integration of the bone grafts. One year later, we decided to re intervene the patient to perform a formal shoulder joint replacement to improve functional outcomes. The operation was carried out successfully and after one year the patient has improved his functionality, being capable of performing full ad/abduction, extension and flexion of the shoulder, elbow and wrist joints. Sensibility remains partially compromised, with intermittent disesthesias over the area covered by the flap, but there are no signs of atrophic changes over the arm and forearm muscles (Fig. 4).

## 3 Discussion

Complex lesions of the upper extremity used to be limited to the clinical scenario of war and combat injuries; Unfortunately, they have progressively become more frequent in civilians, also affecting the pediatric population due to increasing rates of criminality; This represents an alarming problem world wide, carrying significant incidence, mortality and costs for health systems. Pediatric gunshot wounds require an operative intervention in 52.9% of cases, and several factors including high Injury Severity Scores (ISS), have been associated to increased mortality in this population [2].

Treatment approach of this injuries needs to remain multidisciplinary at all points, as relevant decisions need to be taken on a minute-to minute basis, and can affect the definitive outcome of the patient.

Attention should be paid to focus all initial efforts to appropriately performing life saving maneuvers, such as hydric reanimation and stopping the bleeding, even at the expense of compromising the extremity; [3] As delays in this so called “Golden Hour” of trauma can result in an irreversible phase of hypovolemic shock and death. The initial approach of this patients commonly relies over emergency medicine specialists, but all the involved professionals must be made available promptly, as time sensitive situations may arise at all points, including the presence of acute limb ischemia, compartmental syndrome, and the need of a formal replantation procedure. Once stability has been achieved, the main question is whether an extremity saving procedure or amputation should be performed. To appropriately address this issue several factors need to be considered, and the decision should be taken on an individual basis, as to this date, none of the currently available “severity” scales, such as, MESI, PSI, HFS, MESS, LSI or NISSA have been validated for decision making in prospective trials for open population nor have shown any benefit in the treatment of this patients [4,5].

LEAP (Lower Extremity Assessment Project) was an initiative published by Bosse et al. in 2001 which compared five different scales for decision making in complex extremity injuries, and concluded that low scores on any of the scales could correlate with good prognosis after extremity preserving procedures; However, higher scores were not useful to identify patients that would benefit from amputation [6].

Patient comorbidities, age, status of soft tissues (muscular, nervous and vascular), timing, mechanism and personal decision need to be taken in consideration [7] specially in the context of requiring a complex procedure with high probability of failure such as a formal replantation for extensively contaminated wounds. As in this case, opting for amputation on an early manner can represent equal functional prognosis after proper rehabilitation [8].

If an extremity preserving procedure is decided, it should be performed as early as possible, preferably during the first intervention or shortly after, as this has resulted in better results for this patients ; Specially considering the probability of requiring a microsurgical intervention, where delays will result in posttraumatic vasculature, which will importantly affect the outcomes of flap integration.

## 4 Conclusion

While performing an extremity saving procedure, several resources conform the repertoire of reconstructive specialists, but care should be taken on maintaining some basic principles, aiming to cause the less possible morbidity to the patient. Dorsoepigastric flap, is a resource that has been primarily utilized in the setting of mammary reconstruction; It is a variant of the commonly used latissimus dorsi flap, that utilizes only the descending branch of the thoraco dorsal artery, and based on its perforator branches, allows for the creation of an ample cutaneous island while preserving latissimus dorsi [[9], [10], [11]]. Its use has merely been described in complex upper arm reconstruction before, and should be considered an adequate resource, specially in younger patients to avoid the morbidity of more morbid approaches when feasible.

## Sources of funding

The expenses of this article were paid by the authors. There was no additional funding or sponsorship to this research.

## Ethical approval

The publication of this case report was exempt from approval from the ethical board of our institution.

## Consent

Written informed consent was obtained from the patient´s parents for publication of this case report and accompanying images. A copy of the written consent is available for review by the Editor-in-Chief of this journal on request”.

## Registration of research studies

This case report implies no human research. Thus, our article was not registered in this databases.

## Guarantor

Jorge Humberto Rodríguez Quintero is the guarantor of this study.

## Provenance and peer review

Not commissioned, externally peer-reviewed.

## CRediT authorship contribution statement

**Gustavo Jimenez Muñoz-Ledo:** Conceptualization, Methodology, Validation, Formal analysis, Investigation. **Marcos Melgosa-Juárez:** Data curation, Writing - original draft, Visualization, Investigation, Resources. **Julio Palacios-Júárez:** Supervision, Validation, Writing - review & editing, Supervision, Project administration. **Jesus Morales-Maza:** Conceptualization, Resources, Methodology, Validation, Formal analysis, Investigation. **Jorge Humberto Rodríguez-Quintero:** Data curation, Writing - original draft, Writing - review & editing, Software, Validation.

## Declaration of Competing Interest

There is no conflict of interest to disclose.
